# Supplementary material for: A First Assessment of Carbon Nanotubes Grown on Oil-Well Cement via Chemical Vapor Deposition
Source: Nanomaterials (Basel). 2022 Jul 9;12(14):2346. doi: 10.3390/nano12142346 (PMC9317604; doi:10.3390/nano12142346)
Supplement: Supplementary file 1 [file nanomaterials-12-02346-s001.zip › nanomaterials-1785519-supplementary.pdf]

nanomaterials

MDPI

Supplementary information

A First Assessment of Carbon Nanotubes Grown on Oil-Well Cement via Chemical Vapor Deposition

Luca Lavagna [1,2,*], Mattia Bartoli [2,3], Simone Musso [1,4], Daniel Suarez-Riera [5], Alberto Tagliaferro [1,2,6] and Matteo Pavese [1,2]

1 Department of Applied Science and Technology, Politecnico di Torino, C.so Duca degli Abruzzi 24, 10129 Turin, Italy; simone.musso@polito.it (S.M.); alberto.tagliaferro@polito.it (A.T.); matteo.pavese@polito.it (M.P.)
2 National Interuniversity Consortium of Materials Science and Technology (INSTM), Via G. Giusti 9, 50121 Florence, Italy; mattia.bartoli@iit.it
3 Center for Sustainable Future Technologies @POLITO, Istituto Italiano di Tecnologia, Via Livorno 60, 10144 Turin, Italy
4 Massachusetts Institute of Technology, Department of Civil and Environmental Engineering, 77 Massachsetts Ave., Cambridge, MA 02139, USA
5 Department of Structural, Geotechnical and Building Engineering, Politecnico di Torino, C.so Duca degli Abruzzi 24, 10129 Turin, Italy; daniel.suarez@polito.it
6 Faculty of Science, Ontario Tech University, 2000 Simcoe Street North, Oshawa, ON L1G 0C5 T, Canada
* Correspondence: luca.lavagna@polito.it; Tel.: +39-0110904598

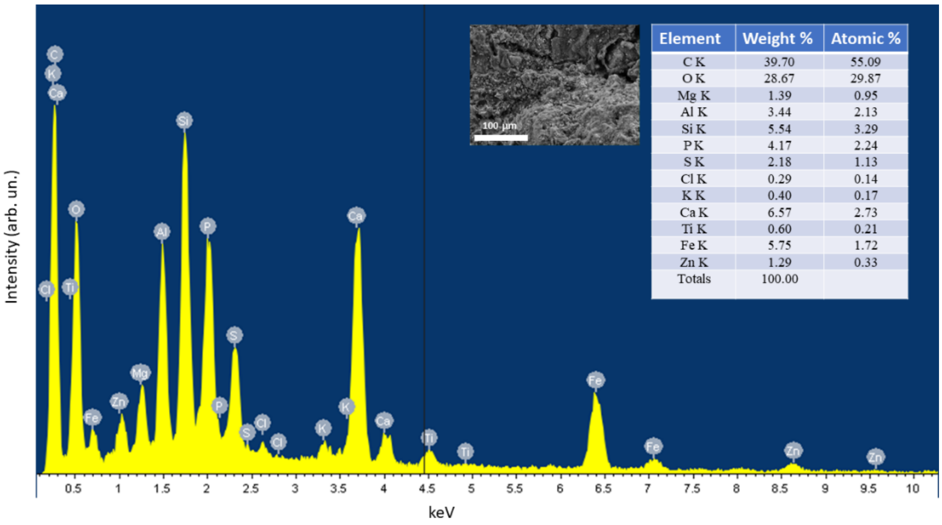

Element	Weight %	Atomic %
C K	39.70	55.09
O K	28.67	29.87
Mg K	1.39	0.95
Al K	3.44	2.13
Si K	5.54	3.29
P K	4.17	2.24
S K	2.18	1.13
Cl K	0.29	0.14
K K	0.40	0.17
Ca K	6.57	2.73
Ti K	0.60	0.21
Fe K	5.75	1.72
Zn K	1.29	0.33
Totals	100.00	

Figure S1: Energy-dispersive X-ray spectrometer (EDS) results of CNTs grown on cement clinker
